# How range shifts induced by climate change affect neutral evolution

**DOI:** 10.1098/rspb.2008.1567

**Published:** 2009-02-25

**Authors:** G.J. McInerny, J.R.G. Turner, H.Y. Wong, J.M.J. Travis, T.G. Benton

**Affiliations:** 1 Computational Ecology and Environmental Sciences, Microsoft Research Limited7 JJ Thomson Avenue, Cambridge CB3 0FB, UK; 2 Centre for Ecology and HydrologyHill of Brathens, Banchory, AB31 4BW, UK; 3 Institute of Integrative and Comparative Biology, Faculty of Biological Sciences, University of LeedsLeeds LS2 9JT, UK; 4 Institute of Biological Sciences, Zoology Building, University of AberdeenAberdeen AB24 2TZ, UK

**Keywords:** climate change, spatial dynamics, gradient, mutation surfing, metapopulation, drift

## Abstract

We investigate neutral evolution during range shifts in a strategic model of a metapopulation occupying a climate gradient. Using heritable, neutral markers, we track the spatio-temporal fate of lineages. Owing to iterated founder effects (‘mutation surfing’), survival of lineages derived from the leading range limit is enhanced. At trailing limits, where habitat suitability decreases, survival is reduced (mutations ‘wipe out’). These processes alter (i) the spatial spread of mutations, (ii) origins of persisting mutations and (iii) the generation of diversity. We show that large changes in neutral evolution can be a direct consequence of range shifting.

## 1. Introduction

The intellectual motivation for ecological and evolutionary research is to explain the distribution and regulation of biodiversity (Elton 1958; Gaston 2003). Many studies suggest that climate change will induce large-scale changes in the spatial distribution of species (e.g. Parmesan 2006). However, far fewer studies consider the mechanisms underpinning range-shifting dynamics (Travis 2003; McInerny *et al.* 2007), and even fewer the evolutionary consequences (Desai & Nelson 2005).

During climate change, species may not simply track suitable climate. At the leading limit of the range, newly available habitat may not be colonized immediately, producing a ‘colonization lag’ (Davis 1989; Mustin *et al.* 2009) and generating a deformation of the range (Rapoport 1975). The process of repeated colonizations into newly available habitat can affect the strength of genetic drift by compounding founder events. This process has been dubbed ‘mutation surfing’ (Klopfstein *et al.* 2005) and can lead to genetic variants attaining a disproportionately wide distribution and high frequency (Excoffier & Ray 2008). Mutation surfing can also apply to those mutants with positive or even negative selection coefficients (Travis *et al.* 2007). Theoretical (Edmonds *et al.* 2004; Klopfstein *et al.* 2005; Wei & Krone 2005; Currat *et al.* 2006; Travis *et al.* 2007; Burton & Travis 2008*a*,*b*; Hallatschek & Nelson 2008) and microcosm (Hallatschek *et al.* 2007) studies investigating the mutation surfing process have investigated invasion in homogeneous environments.

While it is possible to make an analogy between species *range shifting* in response to climate change and those *invading* pristine habitat, there are several distinctions between the spatial dynamics of these two systems. (i) Spatial variation in climate determines the range of many species (Gaston 2003), producing ranges of finite size, whereas, in invasion models, the environment is homogeneous and the population size increases during invasion events. As mutation surfing can alter diversity patterns during invasion (Austerlitz *et al.* 1997; Hallatschek & Nelson 2008), there could be strong differences in the effect of founder events in populations growing in size versus those of stable size. (ii) The processes producing finite ranges also produce heterogeneous patterns of population turnover (Lennon *et al.* 1997). Importantly, the patterns of turnover do not run parallel to occupancy patterns (Antonovics *et al.* 2006). Evolutionary dynamics are sensitive to repeated extinctions and colonizations (Hastings & Harrison 1994), suggesting that non-uniform alterations to turnover, caused by climate change, may produce non-uniform changes in evolutionary dynamics. (iii) Invasion models have focused on an expanding range edge. Species tracking changing climate also have a trailing range limit to consider, where survival will reduce as habitat suitability declines.

To summarize, invasion models are typically models of growing populations, where the dynamics of the range edge are simply a transient dynamic of population parameters (e.g. survival) that are constant across the whole range. Models incorporating climate have (quasi-)stable populations, where parameters vary across the range owing to changes in habitat suitability. Because of these principal differences between range shift and invasion, we developed a model of a metapopulation to investigate neutral evolution during climate change.

Our species is modelled as a metapopulation using a spatially explicit, stochastic model developed to investigate ecological dynamics at range limits (Lennon *et al.* 1997; Holt & Keitt 2000; Antonovics *et al.* 2006). This model benefits from detailed knowledge of the ecological dynamics in static climates, allowing a mechanistic understanding of the spatial processes contributing to evolutionary dynamics. Climate is represented as a change in habitat suitability across space that affects the extinction rate of demes. This is unlike previous studies, which investigated mutation surfing during invasion into homogeneous environments (e.g. Klopfstein *et al.* 2005) or where resources were not replenished (Wei & Krone 2005). Implementing gradients in carrying capacities, in models similar to that of Klopfstein *et al.* (2005), may restrain important spatial dynamics that would otherwise develop (e.g. lags) and the effects may have more similarity to those generated by a landscape feature.

We aim to investigate how range shifting alters the structure of ranges and how changes in survival affect neutral evolution during climate change. Having done this, we further investigate how range shifting will affect patterns of neutral diversity across the metapopulation's range. Our model takes a strategic approach to understanding neutral evolution during range shifting, addressing differences in the spatial structure produced along a gradient described above.

## 2. Material and methods

On a cellular lattice, each cell is occupied or unoccupied by a deme. In each generation, occupied cells become extinct with probability *E*, and empty cells are colonized with probability *C*. We model *C* as a function of the number of propagules present in a cell. Each deme within the metapopulation is considered a single individual and produces *τ* offspring, asexually, in each time step (*τ*=3 in all simulations), whose dispersal is determined by one of two dispersal functions. Dispersal may be local (nearest four neighbours) or follow a wider-ranging geometric function EXP(−*ρd*) (Lennon *et al.* 1997), where *ρ* modifies the dispersal kernel's shape and *d* is the distance in cells with latitudinal and longitudinal movements. The colonization probability is given by

(1) C=1−1/(1+ωj),

where *j* is the number of propagules entering an unoccupied cell and *ω* modulates the effect of *j* on *C*. The value of *ω* is constant through space (*ω*=5 in all simulations). Offspring arriving in cells already occupied by a deme are ignored. If colonization occurs, a single one of the *j* propagules is randomly selected as the colonizer. Demes produce propagules after extinction and the resulting colonizations are immediately calculated before the next extinction event occurs. This simplification of invasion models retains the spatial processes demonstrated previously (figure A1 in the electronic supplementary material). As we assume that population sizes in cells are either 0 (unoccupied) or 1 (occupied), there is no simulation of population growth or changes in gene frequencies within cells, and a deme's propagule production is from a single parent. This can either be conceived as a simplified model of a metapopulation or a spatially explicit simulation of individuals (e.g. where cells have a carrying capacity of 1). All events occur synchronously throughout the lattice.

**Figure 1:**
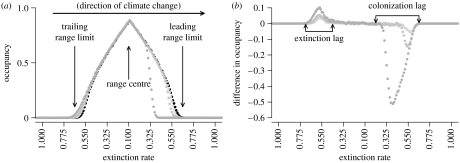
(*a*) Range structure shown in occupancy patterns across the climate gradient, in a static climate (black) and with three intensities of climate change (light grey, *ν*=0.00125; medium grey, *ν*=0.0025; dark grey, *ν*=0.00625; nearest-neighbour dispersal). The shape of these distributions is quasi-stable under the stochastic dynamics. We therefore term these distributions the ‘quasi-equilibrium’ pattern of range occupancy (see text). (*b*) Colonization and extinction lags shown by the difference in occupancy (changing climate−static climate) with increasing rates of climate change. Shadings are the same as in (*a*). Confidence intervals are smaller than plotted points and therefore not shown. Data are taken after 5000 time steps.

We conceive variation in *E* as the relationship between climate and a phenotype, with the minimum value (*E*_min_>0) being the phenotypic optimum. The values of *E* are equal across longitude ([,*y*]), but there is a gradient in *E* applied across latitude ([*x*,]) away from the phenotypic optimum (*E*_min_). From the band of cells initially assigned *E*_min_, extinction probabilities increase linearly, in both latitudinal directions, to 1 (*E*_min=0.1_ in all simulations; see the *x*-axis in figure 1). The linear gradient makes minimal assumptions about the phenotype–climate relationship. Climate change is modelled as a change in the extinction values (habitat suitability) before colonization occurs. *E* increases by *ν* in each time step for cells at latitudes lower than and including the range centre, producing trailing range limits. *E* decreases at the high-latitude side of the range, producing leading limits (where *E*+*ν*>1 values are truncated at 1 and all values are never lower than *E*_min_). The rate of climate change is thus equal across all parts of the range.

Two methods are used to investigate neutral evolution in the asexual and haploid organism. In the first method, the fates of lineages and putative gene flow are tracked through time and space by assigning heritable, unique markers to occupied cells. We monitor the survival and the location of individuals within each lineage. We calculated survival probabilities for *t* generations into the future for a neutral variant initially present at location [*x*,*y*] ([latitude,longitude]). The probability that a lineage will persist (*P*_persistence_), given its initial location [*x,y*] and the time lapsed, is the product of colonization (*P*_colonization_ at [*x*,*y*]) and survival probabilities (*P*_survival_ for *t* time steps from time *T*),

(2) $$P_{\text{persistence}[x,y,T+t]}=P_{\text{colonization}[x,y,T]}\times P_{\text{survival}[x,y,T+t]}.$$

This produces a spatio-temporal distribution of persistence probabilities for the lineages arising along the gradient with *t* time steps elapsed since mutation. Previous studies have mostly investigated the persistence of mutations given the point of origin (but see Hallatschek & Nelson 2008), but to single time points (e.g. Klopfstein *et al.* 2005; Travis *et al.* 2007), masking changes in the temporal distribution of persistence through time. This information is fundamental to understanding diversity patterns.

**Figure 2:**
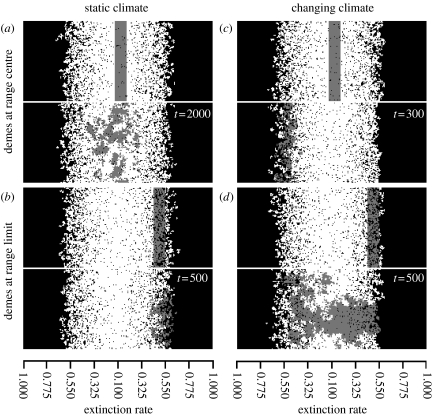
Spatial spread of lineages in (*a*,*b*) static and (*c*,*d*) changing climates (*ν*=0.0025). Demes derived from lineages in (*a*,*c*) the range centre or (*b*,*d*) at the range limit are tracked producing an ecological ‘barium meal’ (§2). The upper section of the panels shows initially marked cells and the lower the metapopulation after the specified period of time. Black cells, unoccupied; white cells, occupied; grey cells, occupied with deme derived from the initial marking. Climate shifts left to right during climate change. Panels aligned at the phenotypic optimum, *E*_min_. Occupancy shown after extinction events have taken place (local dispersal, 300×100 grid). In a changing climate, the range centre would be two cells away from its position at the start of the simulation for every 10 time steps that have elapsed under the climate change rate shown. We align the metapopulation at the phenotypic optimum, which does not change during the range shift. See also figure A2 in the electronic supplementary material for wider ranging dispersal.

Averaging the persistence probabilities over [,*y*] (longitude) gives the probability function along the climate gradient [*x*,] (latitude). We can therefore visualize the geographic spread of lineages within single simulations by displaying the locations of lineages, either individually or grouped (e.g. by latitude), producing an ecological ‘barium meal’ (figure 2). This displays the structure of evolutionary history underlying the metapopulation.

The second method also marks lineages but allows mutations to occur during colonization with rate *μ*. We use an ‘infinite alleles’ assumption, where each mutation is unique and unrepeatable. By holding *μ* constant throughout a simulation, we observe how neutral diversity is regulated by the metapopulation's spatial structure and can test predictions made by the first method.

The metapopulation colonization–extinction processes give rise to quasi-equilibrium dynamics in a static climate. Following a transient period when climate change is initiated, the dynamics settle onto a quasi-equilibrium where there is a stable spatial structure around the moving climate optimum, *E*_min_ (figure 1). The difference between the quasi-equilibria in static and changing climates is similar to the shape of water droplets on flat and tilted surfaces. The rate of climate change affects this quasi-equilibrium, with faster moving climates causing larger colonization and extinction lags (figure 1). Throughout, we summarize the range relative to the climate gradient, rather than to the absolute latitude, [*x*,], as it facilitates comparison between the lineages arising many time steps, and so large distances, apart.

Each simulation run was allowed a very generous ‘burn-in’ period of 5000 time steps, from initializing all cells as occupied, to ensure quasi-equilibrium had been reached. We calculate statistics describing the ecological dynamics (probabilities of extinction and colonization events; patterns of occupancy) along the gradient, under each parameter set. Rates of colonization and extinction are retrieved during the respective events, with occupancy (proportion of cells occupied), survival and diversity measured after extinction has occurred. The origin of each lineage and its survival was tracked through time. Where patterns of diversity were investigated, the location of all mutants was recorded.

## 3. Results

### (a) Changes in spatial patterns within ranges

Different quasi-equilibrium patterns of occupancy exist in metapopulations inhabiting static and changing climates (figure 1). Static climates produce symmetrical ranges around the optimum climates of the range centre (*E*_min_; figure 1*a*). The quasi-equilibrium during climate change has a colonization lag at the leading range limit. This lag increases with the rate of climate change (figure 1*b*). At the new quasi-equilibrium, the leading range limit will be more aggregated owing to the higher occupancy (figure 1*a*). Extinction lags are also produced at the trailing range limit (figure 1*b*) as the climate shifts relative to the metapopulation and so occupancies are higher relative to the climatic conditions.

We illustrate the power of climate change to alter evolutionary dynamics within the metapopulation using screenshots from example simulations (figure 2). In static climates, the lineages at the range centre remain in the central region and spread towards the range limits (figure 2*a*). Those lineages at or near the range limits do not spread into the range centre, surviving at the range limits, if at all (figure 2*b*). In a changing climate, the lineages at the range centre no longer persist in the locations where they arose but move towards the trailing limit of the metapopulation (figure 2*c*). The lineages derived from the leading range limit spread through the metapopulation (figure 2*d*). The time scales for the spread of lineages are also altered. For example, after 500 time steps, the lineages derived from range limits have hardly moved and have decreased in frequency in a static climate, while during climate change those lineages occupy all latitudes and represent a large fraction of the entire metapopulation. Wide-ranging dispersal reduces the intensity of this effect as some propagules may disperse over the directional flows (figure A2 in the electronic supplementary material), but the principal features remain.

### (b) Changes in survival

**Figure 3:**
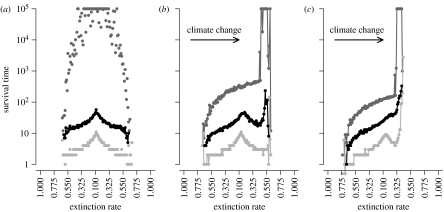
Survival time of lineages arising at locations along the climate gradient in (*a*) static and (*b*,*c*) changing climates (*ν*=0.0025 and 0.00625, respectively). Note that the lineages may survive in locations that are different to their origin (see text). Light grey, median survival times; black, upper 10% survival times; dark grey, maximum survival times. All cells are uniquely marked and lifetimes measured. Increasing *ν* reinforces the change in pattern. Simulations are limited to 10^5^ time steps. Data from tracking 188 734 lineages in a static climate and 187 043 with climate change, both across 20 replicates (200×200 grid, with the gradient occurring over 80 cells in each direction). Lines added to clarify the complex shape of survival times during climate change.

Heterogeneity in survival rates of lineages arising at each location along the gradient is shown in figure 3. In static climates, symmetrical patterns exist, with median survival of lineages originating at the range centre (10 time steps) being far greater than those at range limits (1 time step; figure 3*a*). The lineages originating at the range centre can survive more than 1000 times longer than those from range limits. Dispersal events resulting in colonization occur with greater frequency down the occupancy gradient, away from the range centre, and towards lower occupancy. More propagules are also produced at the range centre owing to the higher occupancy. When combined with smaller extinction probabilities, the lineages from the range centre are expected to have a large contribution to future generations.

Climate change disrupts these patterns. The maximum survival time for any of the statistics plotted is now found just behind the leading range limit (figure 3*b*,*c*), and survival is reduced at the phenotypic optimum. Some of the qualitative features found in static climates are preserved, such as a median survival time of 10 time steps at the range centre, but iterated founder effects during climate change produce median survival times of 100 times greater at leading range limits (figure 3*c*). The lineages arising behind the range centre have reduced survival for higher rates of climate change.

### (c) Changes in mutation generation and persistence

**Figure 4:**
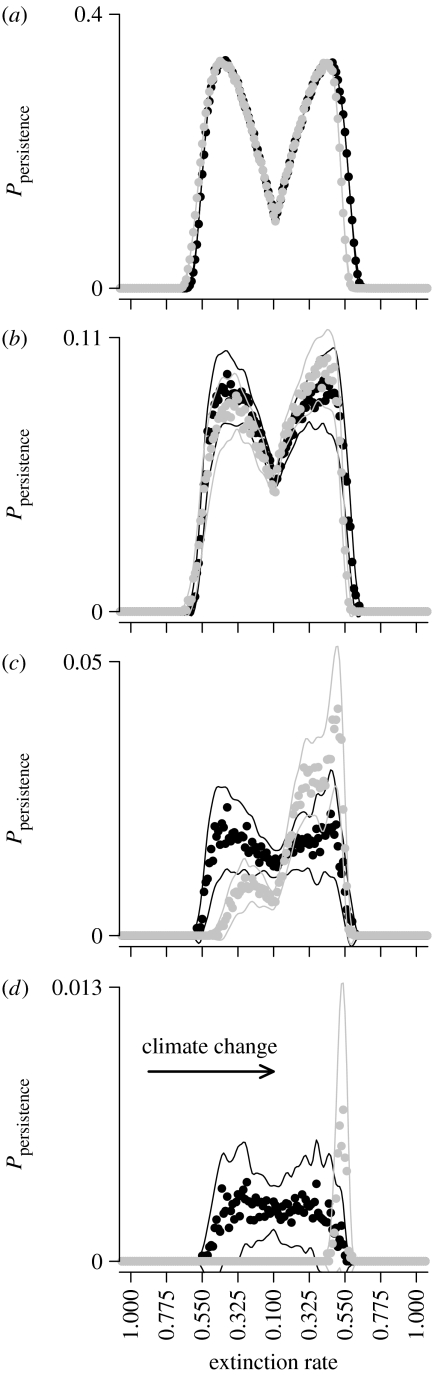
Probabilities describing persistence of lineages (equation (2)) derived from mutations at each location along the gradient, for static (black) and changing (grey) climates (local dispersal). Initially ((*a*) *t*=0), the presence of new mutants is determined by the probability of colonization. Lines show ±1 s.d. in each case (spline, d.f.=60 applied for visual clarity; R project). (*b*) *t*=10, (*c*) *t*=10^2^ and (*d*) *t*=10^3^. Data are the same as in figure 3 (all parameters as in figure 2, except *ν*=0.0025). Equivalent figures for all scenarios and dispersal modes are given in figure A3 of the electronic supplementary material.

In our model, mutation only occurs during a colonization event. Therefore, the colonization dynamics of the metapopulation (‘spatial substructure’; Antonovics *et al.* 2006) determines the patterns of mutation input. The combination of this effect and variation in lineage survival across the metapopulation is illustrated in figure 4 (equation (2)). Colonization events occur nonlinearly through space owing to nonlinear colonization probabilities with changes in *j* (equation (1)), which depends on local patterns of occupancy and nonlinearity in the numbers of empty cells (figures 1*a* and 4*a*; see also Lennon *et al.* 1997). Thus, colonization rates are greatest between range centre and range limits (at *t*=0, no lineages have died; figure 4*a*). Low extinction probabilities at the range centre create few unoccupied cells. At range limits, high extinction probabilities create space, but low occupancy produces few propagules and so colonizations. In between, space is created by frequent extinctions and the occupancy levels produce numerous propagules, causing colonization rates to peak (Antonovics *et al.* 2006). For local dispersal (figure 4*a*), colonization lags at leading limits and extinction lags at trailing limits are visible where there is no overlap of probabilities in static and changing climates. These lags are less apparent with wider-ranging geometric dispersal (figure A3 in the electronic supplementary material).

In a static climate, mutations are likely to occur where colonization rates are highest and most likely to persist when the survival probabilities associated with that origin are subsequently greatest. Thus, persistence for mutations arising at the range centre increases over time in a static climate. In changing climates, increased survival at the leading limit (figure 3*b*,*c*) coincides with high colonization probabilities (figure 4*b*,*c*). This association increases the probability that mutations will occur and persist for significant periods of time within the metapopulation. Towards trailing limits, survival is reduced, producing a strong asymmetry in the expected success of mutants. In the electronic supplementary material, we also show that wide dispersal reduces the coupling of survival and extinction as the colonization lag is reduced, reducing the strength of founder effects (figure A3 in the electronic supplementary material). The variation in the probabilities changes over time, but at different rates across space. Importantly, in a changing climate, variation in persistence increases at a greater rate over time where persistence probabilities are highest (figure 4*d*).

### (d) Changes to the regulation of genetic diversity

**Figure 5:**
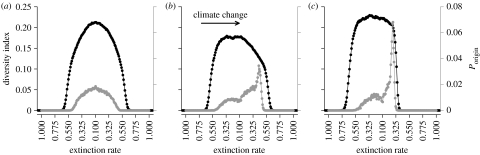
Latitudinal patterns of diversity (black circles and black axis) and probability of surviving mutations having origins at each latitude (grey circles and grey axis) for metapopulations inhabiting (*a*) static and (*b*,*c*) changing climates (*ν*=0.00125 and 0.00625, respectively). Mutation to unique alleles (i.e. an infinite alleles model) occurs with *μ*=0.001. Diversity index is given as the fraction of all extant and distinct lineages contained at each point of the gradient (note that the entire diversity contained under each climate change treatment differs). Data are from a single sample after quasi-equilibrium is reached in each of more than 560 replicates. Other parameters are the same as in figure 4. See figure A4 in the electronic supplementary material for rates of loss of diversity.

Section 3*c* showed that (i) a homogeneous mutation rate at the deme level would lead to nonlinear patterns of mutation generation at the metapopulation level owing to non-uniform rates of turnover, (ii) subsequent persistence of mutations is dependent on their geographic origins, and (iii) the relative importance of mutation generation and survival in persistence changes through time. These factors will affect expected patterns of genetic diversity in simulations where mutation occurs at a constant, positive rate (*μ*>0; figure 5*a*–*c*; see figure A4 in the electronic supplementary material). In static climates, diversity is greatest at the range centre and the ancestors of each lineage originate from the same location (figure 5*a*). With slow climate change (*ν*=0.00125), the greatest diversity is found towards the trailing limit (figure 5*b*). Increasing *ν* homogenizes diversity around the range centre (0.5<*E*<0.3; figure 5*c*). In either case, lineages are more likely to have originated at leading range limits (figure 5*b*,*c*). These patterns are consistent with the survival patterns (figures 3 and 4) and directional flows presented previously (figure 2). The key feature is the mismatch in the locations of origin and survival for extant lineages, which is not present in a static climate.

## 4. Discussion

Our work demonstrates the potential for climate change to alter the spatial dynamics of species and elicit large changes in neutral evolution. Specifically, range shifting can alter (i) the spread of lineages across climate gradients, (ii) the origins of surviving lineages and (iii) the sites of subsequent survival. These three quantities are critical to explaining genetic patterns. Interestingly, we showed that genetic diversity was low at the leading range limit and increased towards the trailing end of a range, with slow climate change, mirroring a pattern frequently found in nature (Hewitt 1996). Within metapopulations, the large effects of spatial relations on evolutionary dynamics have long been appreciated (Wright 1943) and their dominance in determining which processes occur is frequently emphasized (Hanski 1998). This emphasis on spatial pattern is a fundamental feature of our study, where even small changes in the climate change parameter *ν* (equivalent to 1.25% change in phenotypic optimum per time step) produce large changes in neutral evolution. We have characterized the emergent patterns of range shifting, providing insight into the generality of the outcomes as shown by qualitatively similar results across climate change rates and dispersal distances (figures A2 and A3 in the electronic supplementary material).

The dynamics of our model are strongly influenced by iterated founder effects occurring at the leading range limit (mutation surfing; Klopfstein *et al.* 2005). We show an additional effect where survival rates are reduced at trailing range limits (‘wiping out’, within the surfing metaphor). This increases the skew in the distribution of persistence probabilities through a range. This is a non-trivial difference from invasion models, and we suggest that genetic revolutions could occur more rapidly and with greater strength during range shifts. This suggestion is supported by the increased rate of diversity loss during climate change (figure A4 in the electronic supplementary material) and the origins of most surviving lineages being at leading range limits. The radical changes in dynamics are a result of different quasi-equilibrium spatial structures, as characterized by colonization and extinction lags (figure 1). Importantly, these spatial processes are not solely found in small regions of parameter space. The phenomenon is produced by increases in colonization and extinction rates at the respective range limits. Furthermore, in our model, each deme is effectively an individual, demonstrating that the effects are not dependent on changes in dynamics within demes. Colonization lags will be sensitive to reproductive factors such as Allee effects (Keitt *et al.* 2001), and future research should investigate how such mechanisms affect evolutionary outcomes (Hallatschek & Nelson 2008).

Here, introducing simple assumptions appropriate to climate change produces a suite of differences from equilibrium theory or invasion models (e.g. the emergent genetic patterns and magnitude of founder effects can influence the whole metapopulation). There are many other sources of heterogeneity and within-species variation that could give rise to novel outcomes and have already been shown to affect climate change responses, such as landscape structure (Travis 2003; McInerny *et al.* 2007), dispersal evolution (Thomas *et al.* 2001; Simmons & Thomas 2004) and interspecific interactions (Brooker *et al.* 2007). The vast majority of studies investigating the consequences of climate change have so far been cast in an exclusively ecological context, assuming (i) no genetic changes occur during climate change or (ii) genetic changes have no effect on population viability, community assembly or a variety of other interactions. Neither of these assumptions is universally true and genetics may be of considerable importance in many, if not all, ecological processes (Hughes *et al.* 2008).

New theory developed with different methods could offer different insights. We chose a prospective method that investigates the survival of mutations. Retrospective approaches, such as coalescence (Wakeley 2004), may provide some complementary information. However, these approaches can be difficult or even impossible to apply to certain scenarios (Rossindell *et al.* 2008). Our prospective methods can be extended to study adaptive evolution of the species range (Kirkpatrick & Barton 1997). For more tactical applications of the strategic understanding we are acquiring (e.g. Estoup *et al.* 2004), coalescent methods may be particularly useful, not least for their computational efficiency. A clear message from this and previous work (Excoffier & Ray 2008) is that genetic patterns may be strongly influenced by demographic dynamics during changes in ranges (also see Alleaume-Benharira *et al.* 2006). The novel understanding developed here may be incorporated into null models against which tests for selection are made (Hoffman & Willi 2008).

Klopfstein *et al.* (2005) demonstrated links between demographic rates and mutation surfing: surfing was positively correlated with population growth but negatively correlated with carrying capacity and dispersal rate. In invasion models, we would expect these effects as the wave of expansion is a transient. However, in our model, the effects are generated by changes in quasi-equilibrium that will persist throughout climate change. A direct comparison between the results from invasion models (and existing theory; e.g. Otto & Whitlock 1997) and our model is difficult owing to the differences in underlying model structure, i.e. the high degree of spatial structure that emerges in our metapopulation contains. Also, in our model, population growth rates are dependent on the environmental and intraspecific context of a cell. Effective population size declines towards both range limits, but the effects on persistence of a mutation during a range shift are opposite at trailing and leading limits (figure 4). The increased expected persistence at leading limits is contrary to expectations made on the extinction rate that the metapopulation is experiencing in that part of the range (Whitlock 2004). This will undoubtedly alter the persistence of mutations with marginal effects (Travis *et al.* 2007), and so range-shifting effects need to be taken into account when any hypotheses are based on environmental conditions (e.g. extinction rate) or population traits (e.g. effective population size; Crow & Kimura 1970).

While founder effects are not a new concept, studies of this kind demonstrate the importance of iterated founder effects when species are dynamically range shifting across space. During climate change, gene flow into the leading limit is reduced and drift strengthened, which alters our perceptions of sympatry and allopatry within the metapopulation. Novel theory is needed to understand these alterations to the ecological determinants of evolutionary processes during climate change (e.g. Desai & Nelson 2005). This is illustrated by differences in the details found in different scenarios, such as invasion (Edmonds *et al.* 2004), climate change (this study) and Petri dish (Wei & Krone 2005; Hallatschek *et al.* 2007). Importantly, changes in spatial patterning that alter gene flow and drift could then alter the trajectory of evolution (e.g. Kirkpatrick & Barton 1997; Davis & Shaw 2001; Burton & Travis 2008*a*).

## Supplementary Material

Figure A1. Illustration of the surfing phenomenon without a climate gradient

Figure A2. The spread of lineages through space with wider-ranging, geometric dispersal

Figure A3. Persistence probabilities for all parameters investigated

Figure A4. Loss of diversity with and without climate change for local and wide-ranging dispersal
