# Supplementary material for: How range shifts induced by climate change affect neutral evolution
Source: Proc Biol Sci. 2009 Feb 25;276(1661):1527–34. doi: 10.1098/rspb.2008.1567 (PMC2677231; doi:10.1098/rspb.2008.1567)
Supplement: Figure A1. Illustration of the surfing phenomenon without a climate gradient [file rspb20081567s11.pdf]

**Electronic Supplementary Material**

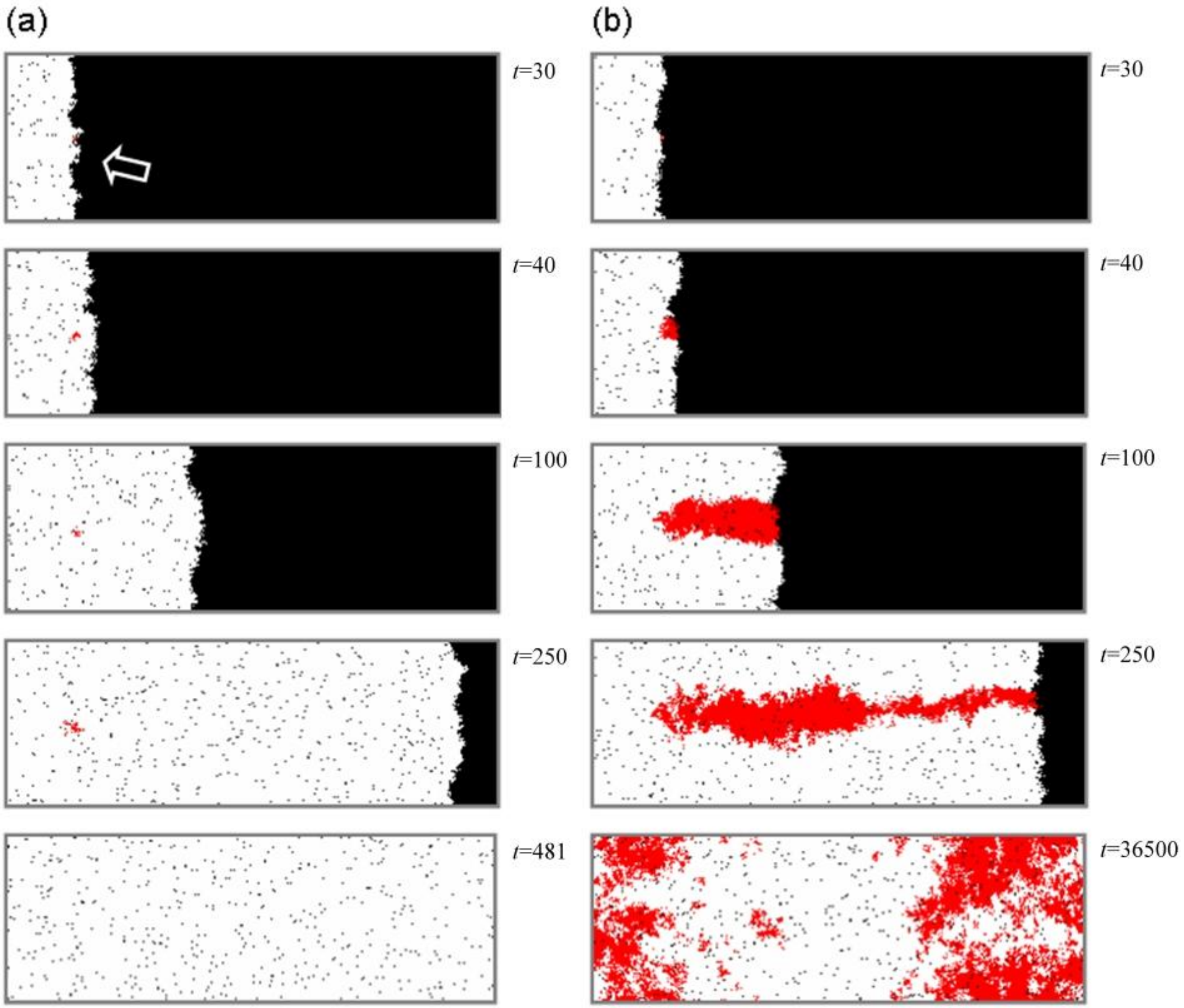


Figure A1: An illustration of the surfing phenomenon described by Edmonds et al. (2004) & Klopfstein et al. (2005). The panels show the invasion of a metapopulation into a pristine landscape (white cells, occupied; black, unoccupied) for 4 time points (*t*= 30, 40, 100, 250). The metapopulation occupies the 10 columns of cells at the left hand side of the habitat until quasi-equilibrium is reached. Thereafter, the whole landscape is available. As the metapopulation invades it is seeded with a neutral mutation (red cells) once it reaches the 40th column along the landscape. This occurs around *t*=30 (an arrow indicates the locations of mutants in (a)) and allows a steady ecological state to form at the expanding range limit. As shown in (a) the mutation may not catch the wave and its ancestors persist within the stochasticity of drift. The individuals at *t*=250 that carry the mutation have spatial locations very close to where the ancestor originated. In (b) the mutation surfs the expanding range limit, with the compound effects of iterated founder effects producing a wider distribution of the mutant. The mutants can occur at large distances from the ancestor's origin. The mutants shown in (a) go extinct at *t*=481. However, a mutant that *surfs* may produce a lineage that persists for >100,000 generations (b) and produce patterns that persist and deform through time (also see Ibrahim et al. (1996)). Many mutants *wipe out* before receiving the *surfing* effect (not shown). Other parameters: $E_{min}$=0.1, $\omega$ =5, local 'nearest-neighbour' dispersal, 300x100 grid.
