# Supplementary material for: How range shifts induced by climate change affect neutral evolution
Source: Proc Biol Sci. 2009 Feb 25;276(1661):1527–34. doi: 10.1098/rspb.2008.1567 (PMC2677231; doi:10.1098/rspb.2008.1567)
Supplement: Figure A2. The spread of lineages through space with wider-ranging, geometric dispersal [file rspb20081567s12.pdf]

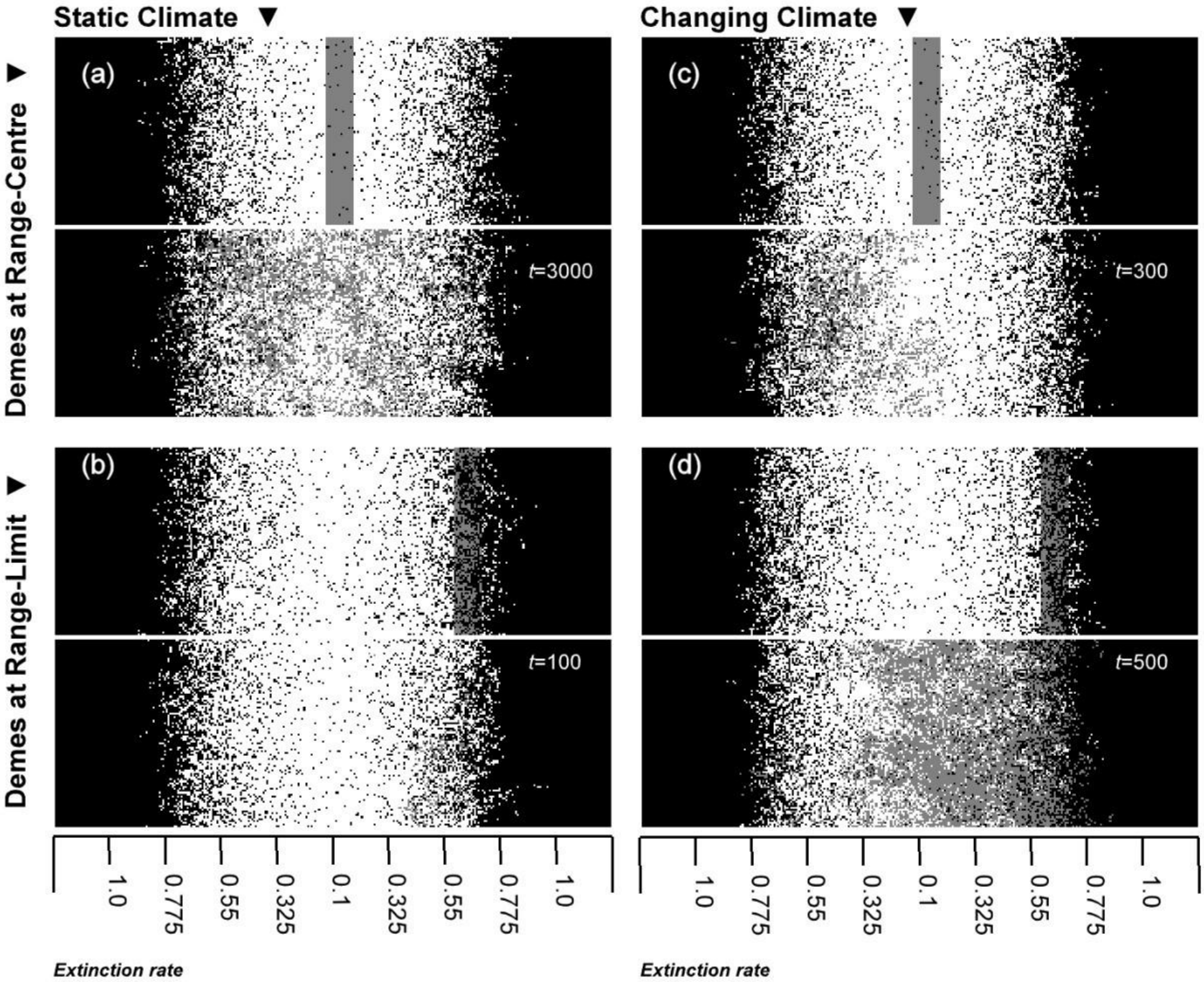

Figure A2: As in figure 2 the spread of lineages in static (a & b) and changing climates (c & d; v=0.0025) but for geometric dispersal (ρ=0.3). Demes derived from lineages in the range-centre (a & c) or at the range-limit (b & d) are tracked producing an "ecological barium meal" (see methods). Upper section of panels show initially marked cells and lower, the metapopulation after the specified period of time. (Black cells, unoccupied; White cells, occupied; Grey cells, occupied with deme derived from the initial marking). Climate shifts left to right during climate change. Panels aligned at the phenotypic optimum, E_{min}. Occupancy shown after extinction. (local dispersal, 300 by 100 grid). The range-centre would be 2 cells away from its position at the start of the simulation for every 10 time steps that have elapsed under the climate change rate shown.
