# Supplementary material for: How range shifts induced by climate change affect neutral evolution
Source: Proc Biol Sci. 2009 Feb 25;276(1661):1527–34. doi: 10.1098/rspb.2008.1567 (PMC2677231; doi:10.1098/rspb.2008.1567)
Supplement: Figure A3. Persistence probabilities for all parameters investigated [file rspb20081567s13.pdf]

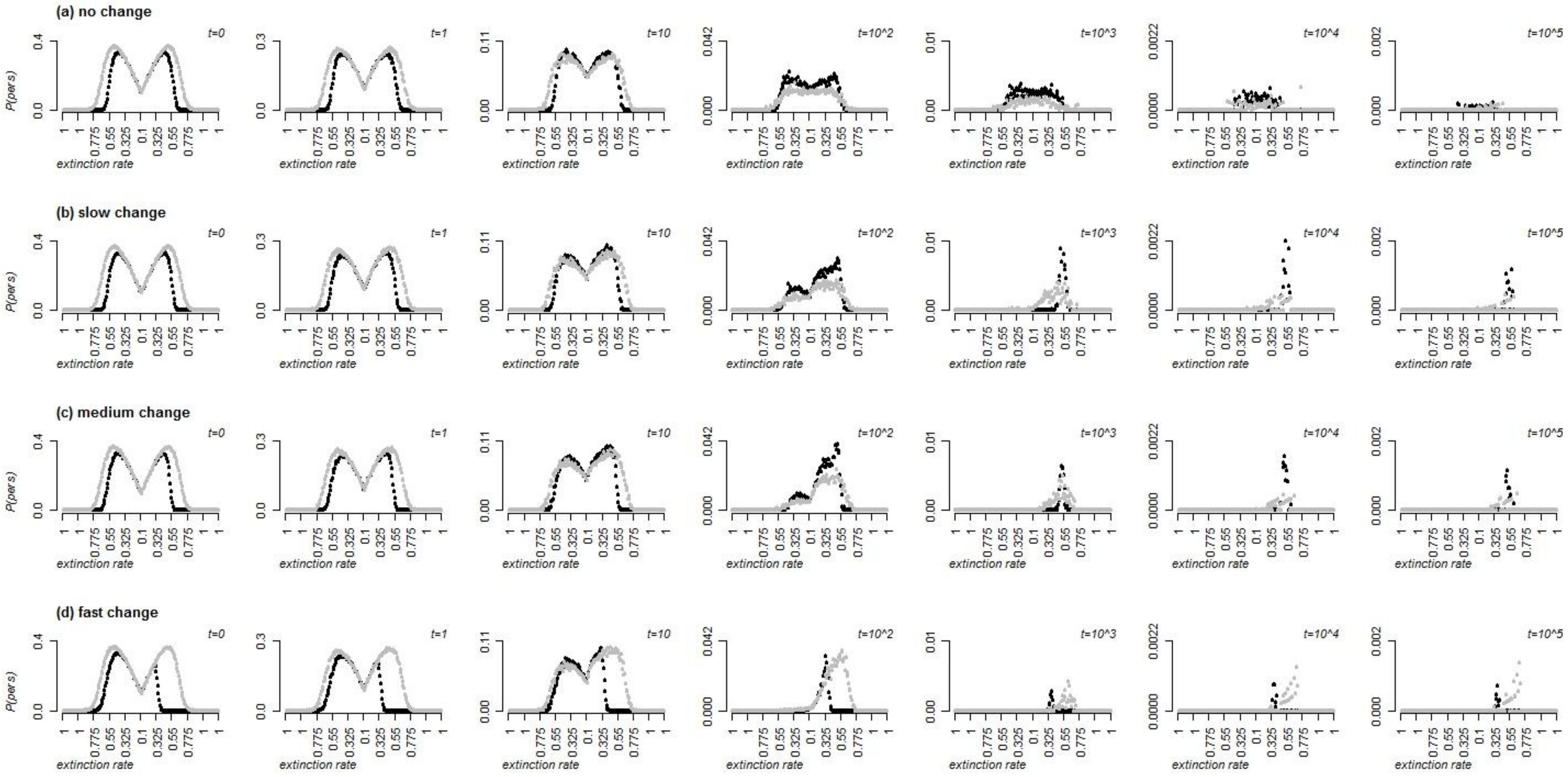

Figure A3: Persistence kernels in a static climate (a) and with three intensities of climate change (b, c & d; v=0.00125, v=0.0025, v=0.00625 respectively). The strength of the *surfing* phenomenon is shown over time and contrasted between local (a) (nearest neighbour dispersal) (black) and wide ranging dispersal (b) (grey) (ρ=0.3). As in figure 3 where t=0 the kernel is equal to the probability of a colonisation event and so can be scaled by a mutation rate to show the probability of a mutation occurring. As t increases the kernel shows the probability that a new colonisation will survive that time into the future, which again may be scaled by a mutation rate, giving the absolute probability of a mutation's occurrence at that point of the gradient and survival to that time step. Parameters as figure 3.
