# Supplementary material for: How range shifts induced by climate change affect neutral evolution
Source: Proc Biol Sci. 2009 Feb 25;276(1661):1527–34. doi: 10.1098/rspb.2008.1567 (PMC2677231; doi:10.1098/rspb.2008.1567)
Supplement: Figure A4. Loss of diversity with and without climate change for local and wide-ranging dispersal [file rspb20081567s14.pdf]

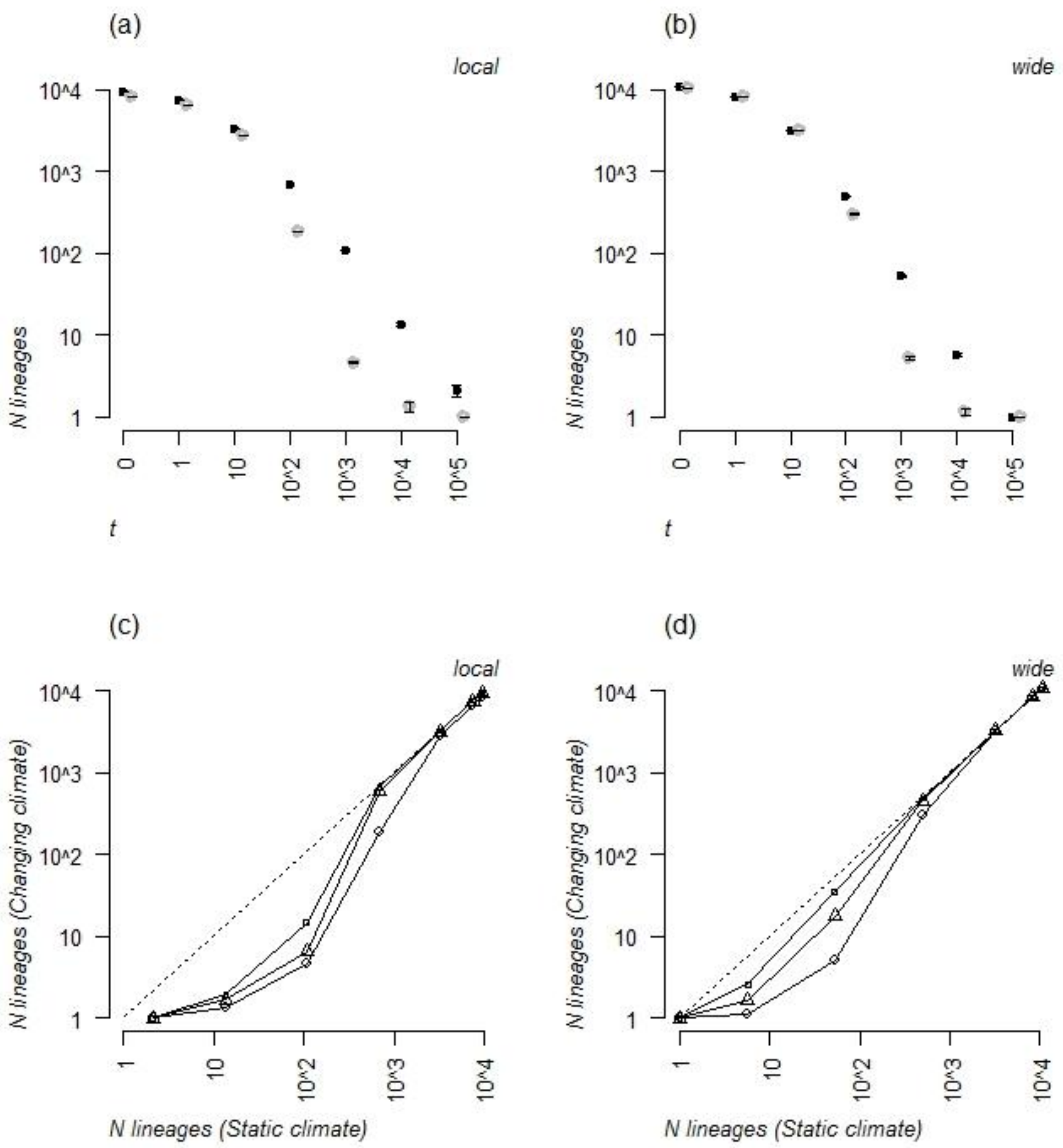

Figure A4: Loss of diversity within static (Black) and changing climates (Grey) for ‘local‘ (a) (nearest neighbour dispersal) and (b) ‘wide’ ranging geometric dispersal (ρ=0.3). Initially all extant cells are marked and non mutations then enter the metapopulation. The climate change rate shown in (a) & (b) is intermediate (v=0.0025). The effect of the rate of climate change are shown for local (c) and wide (d) dispersal distances where the number of lineages at each time point in a static climate (“static” lineages) are plotted against that in a changing climate (“changing” lineages) at the same time step (squares, v=0.00125; triangles, v=0.0025 and circles, v=0.00625). The largest number of lineages are found at g=0, with increasing time the number of lineages decreases. All occupied cells at t=0 are marked as individual lineages and the average number of survivors (10 replicate simulations) are shown at each time step. All other parameters as previously presented. Jitter in x-axis values added for clarity in a & b.
